# Correlates of hepatitis B awareness and disease-specific knowledge among pregnant women in Northern and Central Uganda: a cross-sectional study

**DOI:** 10.1186/s41124-018-0043-6

**Published:** 2018-12-19

**Authors:** Joan Nankya-Mutyoba, Jim Aizire, Fredrick Makumbi, Lynn Atuyambe, Ponsiano Ocama, Gregory D. Kirk

**Affiliations:** 1 Department of Epidemiology & Biostatistics, School of Public Health, Makerere University College of Health Sciences, P.O. Box 7072, Kampala, Uganda; 2 Department of Epidemiology, Johns Hopkins Bloomberg School of Public Health, Baltimore, Maryland USA; 3 Department of Community Health & Behavioral Sciences, School of Public Health, Makerere University College of Health Sciences, Kampala, Uganda; 4 Department of Medicine, School of Medicine, Makerere University College of Health Sciences, Kampala, Uganda; 5 Department of Medicine, School of Medicine, Johns Hopkins University, Baltimore, Maryland USA

**Keywords:** Hepatitis B, Awareness, Knowledge, Pregnant Women

## Abstract

**Introduction:**

Countries in sub-Saharan Africa with a high hepatitis B burden also have limited resources to identify underlying drivers of disease among key at-risk populations. To improve prioritization and strengthen prevention of mother to child transmission of HBV, it is imperative to understand disease awareness, knowledge and related factors among pregnant women.

**Objectives:**

This study assessed HBV disease awareness, knowledge and related factors among pregnant women in public health facilities in two regions with diverse HBV disease epidemiology.

**Methods:**

From October 2016 through December 2017, a random sample of 455 pregnant women attending antenatal clinics were surveyed to assess HBV awareness, knowledge and associated factors. Participants responded to an 18-item questionnaire with themes on HBV awareness, knowledge of disease signs and symptoms, transmission, prevention and misconceptions about the disease. Results were analysed in STATA (version 14.0).

**Results:**

Of 455 participants enrolled, about two thirds reported having heard about HBV disease. By region, nearly half (47%) of participants from the central region, compared to only 16% from the north, reported that they had never heard of HBV. Region of residence had a moderating effect on the education- HBV awareness relationship. Only 162/455 (36%) of participants had adequate HBV knowledge. More than half 256/455 (56%) and 242/455 (53%) were not knowledgeable about horizontal and mother to child HBV transmission, respectively. About two thirds 298/455 (66%) and 281/455 (62%) believed HBV was spread via sharing of utensils and mosquito bites respectively. In multiple regression analysis, residing in the north, (PR=1.91(1.53 -2.38), *p* < 0.001) compared to central region and having a secondary education (PR=1.87(1.37 -2.55), *p* < 0.001) compared to primary were statistically significantly related to being knowledgeable about HBV.

**Conclusion:**

We demonstrated marked regional differences in HBV disease awareness and knowledge in this high HBV prevalence setting. However, most pregnant women displayed unacceptably low HBV knowledge and a significant proportion still hold misconceptions about HBV. Interventions to improve HBV prevention through antenatal education will need to be tailored to existing differences in comprehensive HBV knowledge.

## Introduction

Chronic Hepatitis B virus (HBV) infection is among the commonest infections world-wide, with an estimated affected population of over 240 million individuals [1]. Chronic infection with HBV places these individuals at increased risk of death from cirrhosis and liver cancer [2–4]. A significant portion of Africa’s HBV burden is carried in sub-Saharan African (SSA) nations [5]. Uganda has recorded a much higher HBV burden than other countries within the East African sub-region, with national estimates averaging 10% and notable regional variations in disease burden [6, 7]. A recent study using modelling and expert opinion reported Uganda to have an estimated HBV prevalence of 5.5% and 1.5% among adult and 5-year old populations respectively [8]. Surveys conducted in two important sub-populations i.e. health care workers [9, 10] and pregnant women [11] have found HBV infection prevalence above 10%, although an earlier study [12] among HIV-infected pregnant women from a private, not-for-profit hospital had reported hepatitis B surface antigen positivity of 4.9%. It remains uncertain however, whether disease awareness in this population sub-group is adequate and whether variations in knowledge gaps mirror those observed in disease burden. The response to viral hepatitis at national program level in Uganda has been modest [13–16]. Like most SSA countries, routine HBV vaccination of infants before one year is high, at 93%, but only 3% of infected pregnant women eligible for antiviral therapy have been initiated on treatment. Screening of women for HBV during pregnancy, plus follow-up care including full vaccination, treatment with hepatitis B immunoglobulin and birth dose vaccinations yet to be implemented in public health care facilities in Uganda [8]. In addition, there is inadequate research evidence on HBV disease epidemiology and population-level knowledge and awareness. While HBV may be known as infectious and cancer-causing within the medical and scientific community, it remains unclear how much knowledge exists in the general population, to which pregnant women mostly belong. The first global health sector strategy on viral hepatitis by the world health organization (WHO) to eliminate HBV [17] as a major global health threat by 2030, calls for information that is usable for action, including increase in access to HBV vaccinations and general disease awareness in populations. This current gap in HBV research contributes a barrier to health system strengthening for HBV elimination.

Pregnant women remain an important sub-population for prevention of mother to child transmission of HBV [3, 18]. In countries where antenatal HBV prevalence is high, they represent a population sub-group to be targeted for HBV micro-elimination. If pregnant women have accurate information on HBV transmission and prevention through vaccination, they are more likely to engage with the health care system, to actively seek and get testing services and ensure their unborn babies are protected, as studies in similar infectious diseases have shown [19, 20]. Yet there is limited documentation of the extent of awareness and specific knowledge about HBV, its transmission or prevention, among pregnant women. Within SSA, the few studies to evaluate disease awareness and knowledge among pregnant women have been mostly conducted in West [13, 14] and Central Africa [15, 16], which have different cultures and disease burden. We conducted a survey among pregnant women attending routine antenatal clinics in public health facilities in 2 out of 4 regions of Uganda, namely Central and Northern regions, with the objective of measuring hepatitis B prevalence and risk factors and assessing HBV and liver cancer knowledge, perceptions and prevention intentions in this obstetric population. The two regions have different epidemiologic profiles of HBV with prevalence ranging from 6% in the Central region and close to 20% in the Northern region [10]. This analysis focuses on HBV awareness and knowledge.

## Methods

### Study setting

This was a cross-sectional study conducted in public health care facilities in central and northern regions. In the northern region, the study site was Arua Hospital, a regional referral hospital located in Arua town, about 300 miles north-west from Kampala city. It is a high volume, 323 bed-capacity hospital that serves an estimated population of 782,077 covering districts of West Nile and parts of Northern Uganda. It receives about 153,451 out-patients and 5,149 antenatal clinic attendees per year [21]. In the central region, study sites were Kiswa health center III and Kasangati health center IV, both high volume primary care health facilities [22] located in suburban areas in Kampala and Wakiso districts. Both health centers receive 850-1,000 antenatal attendees per month, on average.

### Study population

For inclusion, eligible participants had to be pregnant, attend their routine antenatal clinic visit, have a medical file with the health facility and be at least 18 years of age. Participants who did not provide written informed consent, plus those who could not understand the interviewing language or who were too ill to undergo study-specific procedures were excluded.

### Sample selection

We used Kish & Leslie formula [23] for sample size estimation, for an estimated knowledge prevalence of 50% and a 5% error rate. We included a non-response fraction of 10%, and obtained a total sample size of 455. Participants were sampled using a two stage-sampling approach. In the central region, all health center III and IV facilities were enumerated, and stratified by urban or semi-urban status, the divide based on population served [24, 25]. Then, one health facility was randomly selected from each group, i.e. Kiswa and Kasangati health centers, both of which serve urban and semi-urban populations in different localities of the central region. In the North-west, Arua hospital was selected, considered to serve both urban and semi-urban populations in this region.

Pregnant women were recruited from antenatal clinics on Mondays, Tuesdays and Thursdays in Kiswa and Kasangati health units, and on Mondays and Thursdays in Arua, as these were days when general antenatal clinics operated. All women were generally informed about the study during antenatal education sessions. Then, women to participate were systematically sampled; every 5^th^ woman waiting in line to be seen was approached and informed about the study in detail, and informed consent was sought and obtained prior to study enrollment. Some women preferred to go through the study procedures before proceeding to complete their clinical evaluation, while others preferred to complete study procedures after completion of their routine antenatal visit process.

### Data collection

Trained nurses or midwives were trained as study personnel. They underwent a 3-day training on study-specific procedures, HBV facts, interviewing techniques, ethical issues and data documentation. In addition, they each completed a certified NIH online ethics training course. One of the training days was used to pilot study tools, using clinic nurses and pregnant women from nearby health units that would not be used for data collection. Questionnaires were refined according to feedback received.

Questionnaires were administered to every consenting woman to document data on HBV and liver cancer awareness and knowledge, perceptions and preventive behavioral intentions. Questions on knowledge inquired about whether participants had ever heard of HBV disease, its signs and symptoms, and body part affected. Questions on routes of transmission asked whether participants knew that HBV is transmitted sexually, or from mother to child during child birth, or by sharing of needles, or by contact with infected body fluids, through practices like oral warming or pre-chewing of children’s food. Other questions asked about routes of transmission that have no bearing to HBV. Questions on prevention asked whether participants knew that HBV or liver cancer can be prevented with a vaccine. Information on socio-demographic factors (age, religion, marital status, highest education achieved, region where one was born, region where one has lived for past 12 months) was also obtained from participants.

### Measures

#### Hepatitis B disease awareness

We assessed HBV disease awareness using two approaches. First, using one question “*Have you ever heard of a disease called hepatitis B?*” (response as “Yes”/“No”), the proportion of participants who responded “Yes” and “No” were classified as being “aware” and “Unaware” of HBV disease, respectively. In the second approach, to further measure disease awareness, we inquired from those participants who responded “yes” to the question “*Have you ever heard about hepatitis B?*”, 3 more questions; “*Are you aware of which body organ gets affected by hepatitis B? Are you aware of any sign or symptom of hepatitis B? and are you aware of any route by which hepatitis B may be spread?*”, to which participants had to mention a body part affected, a sign or symptom and one route or method of disease spread, respectively and responses were documented. Participants with correct responses to at least two of the three questions were then further classified as having “high awareness”, else they were classified as having “low awareness”.

#### Knowledge of HBV

In order to further evaluate for more comprehensive HBV disease knowledge, participants were asked 10 questions which inquired about HBV transmission routes (4 questions), prevention (2 questions) and common misconceptions (4 questions) about the disease and its causation. For each question item, responses were either “Yes/No/Don’t know” or “True/False/Don’t know”. Each correct response was assigned a score of ‘1’ while each incorrect response was assigned a score of ‘0’. A composite variable of knowledge scores was generated. Two cut off values of knowledge scores were evaluated for possible cut off for adequate knowledge, 7/10 and 8/10. The cut off score of 7/10 was considered to be justifiable, for the extent of difficulty of the questions and the study population. It was comparable [26, 27], and in some instances more stringent [28] than cut off values of studies in similar populations elsewhere.

A cut off value of 7/10 correct responses was therefore defined as “adequate knowledge” and scores below this value as “inadequate knowledge”. Two questions were not included in the score for knowledge variable, as these were considered as knowledge to be expected from individuals with a medical training, yet the study population were lay persons.

#### Socio-demographic variables

Given the narrow age range, age was classified into three categories of under 20 years, 20-24 years and older than 24 years, according to categorizations of the Uganda demographic and health survey. This categorization also enabled examination of the relationship between adolescent compared to older pregnant women, regarding HBV disease awareness and knowledge. Three nominal categories of religion were created (catholic, protestant and Islam/other). Likewise, region of birth was categorized into three groups, “North”, “Central”, and participant who reported have been born in regions besides these two, were classified as “other”. Individuals born outside the two study regions were few, and these were merged to form the third category. Education was grouped into two, primary and secondary, as very few individuals had post-secondary education. Status of marriage was also categorized into monogamous, polygamous and divorced/single/other. The third category was merged into one group due to very few participants who reported being single, divorced and/or separated.

#### Data analysis

Raw data was entered into excel and data cleaning done before it was exported to STATA (version 14.0) for analysis. Descriptive and stratified analyses were done and data summarized as means with corresponding standard deviation (sd) for continuous variables and proportions for categorical variables. Chi-square for difference in proportions were done. Bivariate analyses were done to estimate associations between individual factors with (i) HBV awareness (ii) HBV knowledge.

Variables that were significant at a *p*-value of 0.20 at bivariate level, plus those considered important based on plausibility were entered into a multivariable model. We used a modified Poisson regression model with robust variance estimation to obtain prevalence rate ratios [29, 30] with 95% confidence intervals, given that the outcomes had a prevalence greater than 10%. In adjusted models for HBV awareness, we estimated the effect of region of residence, on the association between education and HBV awareness. This was estimated from summing up the coefficients of education level and the interaction term, from the multivariable model. The summed up coefficient was then exponentiated to obtain the prevalence risk ratio for this stratum. Education had a moderating effect on the observed relationship between region of residence and HBV disease awareness, which was computed using a similar method. A p-value of 0.05 was used as a cut off for statistical significance. To further evaluate disease awareness and knowledge, we constructed a binary “awareness & knowledge” variable, which was defined as being both aware of, and having comprehensive HBV knowledge as initially defined as a knowledge score of at least 7 out of 10, “adequate knowledge”, or being neither aware of, nor having comprehensive hepatitis B knowledge. Factors associated with the composite “awareness & knowledge” variable were evaluated in both bivariable and multivariable models.

## Results

A total of 455 participants who were approached consented to study participation and completed the questionnaire, 155 from the northern region and 300 from the central region antenatal clinics, giving a response proportion of 100%. About 45 participants opted to complete the survey in English, instead of the local language (*Lugbara*, Northern region and *Luganda*, Central region).

### Pregnant women’s socio-demographic characteristics

The socio-demographic attributes of participants are shown in table 1. The median age was 24 years (IQR=21-28) and 14% of respondents were adolescents in both regions. A higher proportion of participants were from the central region, who contributed 300 (65.9%) of the total sample, while participants from the north contributed 155 (34.1 %) of the sample.

**Table 1:** Socio-demographic characteristics of study participants, overall and according to site of recruitment.

Characteristic		Total *N* = 455	North *N* = 155 (34.1%)	Central *N* = 300 (65.9%)	*p*-value*
Age (years)Median (IQR)		18-45	18-42	18-45	
Age group (years), n (%)	<20	65 (14.4)	22 (14.2)	43 (14.4)	0.477
	20-24	174 (38.4)	54 (34.8)	120 (40.3)	
	>24	214 (47.2)	79 (51.0)	135 (45.3)	
Education level, n (%)	≤Primary	156 (34.7)	57 (36.8)	99 (33.6)	0.496
	≥Secondary	294 (65.3)	98 (63.2)	196 (66.4)	
Region of birth, n (%)	North	136 (30.0)	129 (83.8)	7 (2.34)	**< 0.001**
	Central	168 (37.1)	2 (1.3)	166 (55.5)	
	Other	149 (32.9)	23 (14.9)	126 (42.1)	
Marital status, n (%)	Single/divorced	24 (5.2)	5 (3.25)	19 (6.3)	**< 0.001**
	Monogamy	209 (46.0)	130 (84.4)	79 (26.3)	
	Polygamy	122 (48.7)	19 (12.3)	202 (67.3)	
Religion, n (%)	Catholic	157 (34.5)	66 (42.6)	91 (30.3)	**0.002**
	Protestant	204 (44.8)	52 (33.5)	152 (50.7)	
	Islam/other	94 (20.7)	37 (23.9)	57 (19.0)	

*p-value =chi-square

Two thirds of participants, 294/455 (65.3%), had achieved at least a secondary-level education. Marital unions were reported as mostly monogamous in the north, 130/155 (84.4%), but mostly polygamous in the central region, 202/300 (67.3%) and almost half of participants in the north were catholic, (43.0%) while half of participants in the central region were protestant, 152/300 (50.7%).

### Awareness of HBV

**Fig. 1:**
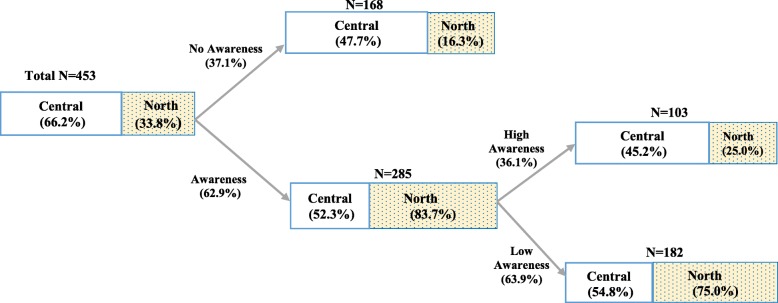
Proportion of women with no awareness, low awareness and high awareness of hepatitis B

Overall, about two thirds, 285/453, (62.9%) of participants had ever heard about HBV disease. By region, only about half, 157/300 (52.3%) of participants from the central region had ever heard of HBV, compared to majority, 128/153, (83.7%) participants from the North. Additionally, among those overall who had ever heard about HBV, more than two thirds, 182/285 (63.9%) had low awareness of HBV. Within the northern region, three quarters of participants, 96/128 (75.0%), had low HBV awareness, versus slightly over half, 86/157 (54.8%) of central region participants (Fig. 1). Only a quarter (25%) of participants from the north had high HBV disease awareness, compared to 45% of those from the central region.

Figure 1 Flowchart showing numbers and proportions of respondents, by geographic region (North and Central) who reported having heard (Awareness) or never having heard (No Awareness) about HBV, plus those who had low and high HBV awareness, among those who reported that they were aware about HBV. Total N=Total number of participants

About half (49%) of adolescent pregnant women reported that they had never heard about HBV, compared to only 35% of the older women. Among pregnant adolescents who reported having heard of HBV, 20% (compared to 43% of older women) had low awareness of HBV. Overall, majority of respondents (77.4%) were either completely unaware of HBV disease, or only heard of the disease, but were unaware of its signs, symptoms or how it is spread.

### Knowledge of HBV

**Fig. 2:**
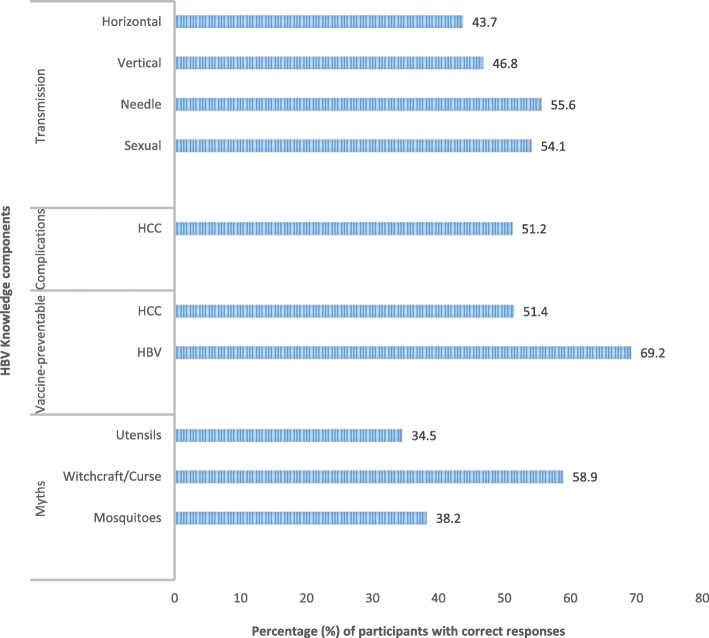
Distribution of proportion of women with correct responses about different hepatitis B knowledge components

Using a defined cut off score for adequate HBV knowledge, only about 36% (162/455) of participants were knowledgeable about HBV, of which 60% (98/162) were from the central region. Figure 2 provides details about components of HBV knowledge (transmission, prevention, complications and myths related to causes). More than two thirds of all participants (69.2%) knew that HBV infection is preventable by vaccination. Approximately half the surveyed population knew that HBV infection can result in liver cancer and that this can be prevented by HBV vaccination (51%), were knowledgeable about sexual (54%) and needle-sharing (56%) routes of HBV transmission. Fewer participants overall, knew that HBV can be spread to the unborn baby by infected mother during child birth (46.8%), or to the newborn via horizontal transmission (43.7%), when newborn may come into contact with blood or body fluids of an infected adult within the household environment. Knowledge about sexual route of transmission was more prevalent in the Northern region (70.3%) compared to the central region, (45.7%) while knowledge about horizontal transmission was more prevalent in the central region,(50%). In both regions, only about half, (Central region 44.7%, Northern region 51.3%) of participants were knowledgeable about mother-to-child transmission of HBV. Regarding misconceptions, a minority of participants knew that HBV is not spread by mosquito bites (38.2%), or by sharing cooking utensils (34.5%) and a sizeable proportion (41.0%) believed the disease can be caused by a curse or witchcraft (Fig. 2).

Figure 2 Proportion of respondents with correct responses to questions on routes of HBV transmission, complications, vaccine prevention and myths regarding causes. HCC=hepatocellular carcinoma or liver cancer. HBV=hepatitis B virus.

### Correlates of HBV disease awareness

In both unadjusted and adjusted models (Table 2.0; model 1 and table 3.0; model 1, respectively) region of residence, age and education were related to HBV awareness. Region of residence had a moderating effect on the observed relationship between education and HBV awareness. Among participants residing in the central region, having a secondary education was associated with 76% greater prevalence of HBV awareness compared to having a primary or lower level of education (PRR=1.76 [95%CI=1.32-2.35] *p* < 0.001), whereas among participants residing in the north, having a secondary education, compared to primary or lower level was associated with only 25% greater prevalence of HBV awareness, adjusting for age (PRR=1.25). Similarly, education had a moderating effect on the observed relationship between region of residence and HBV disease awareness. Among participants with primary or lower level of education, residents of northern region had a prevalence of HBV awareness that was twice that of central region residents (PRR=2.04 [95%CI=1.49-2.80] *p* < 0.001), while among participants with a secondary or higher level of education, residents of the north, compared to central region had only 45% (PRR=1.45) greater HBV disease awareness prevalence, adjusting for age. Pregnant women older than 24 years had a prevalence of HBV awareness that was 33% greater than that of women aged under 20 years (PRR=1.33 [1.03-1.70] *p* = 0.026) and this association was statistically significant. In bivariate analyses, being born in the north compared to elsewhere (PRR=1.48(1.26 -1.73), *p*<0.001) was associated with a prevalence of HBV awareness that was 48% higher. However, this variable highly correlated with region of residence, and was dropped from the multivariable models. Marital status and religion were unrelated to HBV disease awareness.

**Table 2:** Bivariable regression analysis of factors associated with hepatitis B virus awareness and knowledge^

Factor	Model 1^a^			Model 2^b^			Model 3^c^		
	Unadjusted PRR	95%CI	*p*-value	Unadjusted PRR	(95%CI)	*p*-value	Unadjusted PRR	(95%CI)	*p*-value
Age group (years)									
< 20	1			1			1		
20-24	1.19	(0.91 - 1.56)	0.214	1.27	(0.81 - 2.01)	0.302	1.13	(0.76 - 1.66)	0.553
> 24	**1.35**	**(1.04 - 1.75)**	**0.023**	1.41	(0.90 - 2.21)	0.129	1.05	(0.71 - 1.55)	0.804
Education level									
≤ Primary	1			1			1		
≥ Secondary	**1.47**	**(1.23 - 1.75)**	**< 0.001**	**1.40**	**(1.05 - 1.86)**	**0.021**	**1.40**	**(1.05 - 1.86)**	**0.021**
Region of birth									
Other	1			1			1		
North	**1.48**	**(1.26 - 1.73)**	**< 0.001**	**1.91**	**(1.43 - 2.56)**	**<0.001**	**1.42**	**(1.04 - 1.94)**	**0.026**
Central	0.89	(0.72 - 1.09)	0.250	0.97	(0.68 - 1.38)	0.875	1.14	(0.83 - 1.56)	0.427
Religion									
Islam/other	1			1			1		
Protestant	0.90	(0.75 -1.09)	0.283	0.81	(0.58 - 1.13)	0.208	0.88	(0.63 - 1.22)	0.450
Catholic	1.03	(0.85 -1.24)	0.791	1.04	(0.76 - 1.43)	0.808	1.05	(0.76 - 1.46)	0.759
Marital status									
Single/Divorced	1			1			1		
Monogamy	1.37	(0.95 -1.98)	0.091	1.34	(0.71 - 2.54)	0.360	1.03	(0.63 - 1.70)	0.897
Polygamy	0.88	(0.60 -1.29)	0.500	0.67	(0.34 - 1.30)	0.232	0.71	(0.43 - 1.19)	0.198
Residence region									
Central	1			1			1		
North	**1.60**	**(1.41 - 1.82)**	**< 0.001**	**1.97**	**(1.57 - 2.47)**	**< 0.001**	**1.32**	**(1.03 -1.68)**	**0.029**

Model 1^a^*Outcome is “HBV awareness”.* Model 2^*b*^*Outcome is “Adequate HBV Knowledge”.* Model 3^*c*^*Outcome is composite score of “Awareness or Knowledge”* ^Knowledge= A score of at least 7/10. *PRR=Prevalence rate ratio*

**Table 3:** Multivariable regression analysis of factors associated with hepatitis B awareness and knowledge^

Factor	Model 1			Model 2			Model 3		
	Adjusted PRR	(95%CI)	*p*-value	Adjusted PRR	(95%CI)	*p*-value	Adjusted PRR	(95%CI)	*p*-value
Age group (years)									
< 20	1			1			1		
20-24	1.14	(0.88 -1.48)	0.308	1.09	(0.74 -1.61)	0.652	1.22	(0.81 -1.84)	0.349
> 24	**1.33**	**(1.03 -1.70)**	**0.026**	1.04	(0.71 -1.52)	0.856	1.32	(0.89 -1.98)	0.172
Education level									
≤ primary	1			1			1		
≥ Secondary	**1.76**	**(1.32 - 2.35)**	**< 0.001**	**1.39**	**(1.05 - 1.85)**	**0.023**	**1.87**	**(1.37 - 2.55)**	**< 0.001**
Region of residence									
Central	1			1			1		
North	**2.04**	**(1.49 -2.80)**	**< 0.001**	**1.33**	**(1.04 -1.70)**	**0.023**	**1.91**	**(1.53 -2.38)**	**< 0.001**
Education*Residence	**0.71**	**(0.51 - 0.99)**	**0.048**	---	---		---	---	

Model 1*Outcome is “HBV awareness”. Model 2**Outcome is “Adequate HBV Knowledge”. Model 3***Outcome is composite score of “Awareness or Knowledge” ^Knowledge= A score of at least 7/10.

### Correlates of HBV knowledge

Prevalence of adequate HBV knowledge, defined as a knowledge score of at least 7/10, was 35.5% (162/455). In both unadjusted and adjusted models, region of residence and education were related to having comprehensive knowledge of HBV. In multivariable models, participants residing in the northern region had a statistically significant 33% increase in prevalence of adequate knowledge (PRR=1.33 (1.04 -1.70), *p* = 0.023) compared to those in the central region. Pregnant women with a secondary or higher level of education had a statistically significant 39% increase in prevalence of adequate comprehensive knowledge of HBV (PRR=1.39 (1.05 -1.85), *p* = 0.023), compared to women with a primary or lower level of education. Age, religion, and marital status were not related to having comprehensive HBV knowledge. Region of birth was dropped from the models due to correlation with region of residence, as most participants tended to reside in the same region they were born. In models without region of residence, region of birth was statistically significantly related to comprehensive HBV knowledge. Factors associated with the composite “awareness & knowledge” variable are shown in Table 2.0, model 3 and table 3.0, model 3. Region of residence (PRR=1.91(1.53 -2.38), *p* < 0.001) and education (PRR=1.87(1.37 -2.55), *p* < 0.001), emerged as significantly related to being aware and knowledgeable about HBV. There was no significant interaction between education, region of residence and being aware of or having HBV knowledge.

## Discussion

We found a low level of HBV disease awareness with pregnant adolescents tending to have lower HBV awareness, and even lower levels of adequate HBV and liver cancer knowledge in this obstetric population of a high HBV prevalence country, which also records one of the highest HBV-associated liver cancer rates in the world [31]. Higher education, and residing in the northern region, compared to the central region, were associated with being knowledgeable about HBV. Awareness measured using a single question was moderate, but further assessment of awareness revealed that a majority of respondents had low awareness of HBV signs or symptoms, body part mostly affected and at least one method by which the disease spreads. The WHO global health sector strategy on viral hepatitis [17] recognizes the need for critical, indigenous information to direct prevention interventions in different localities and to utilize opportunities like prevention of mother to child HBV transmission through antenatal testing of pregnant women. A key barrier to achieving these goals is low population awareness of HBV, with under 5% of chronic hepatitis-affected persons aware of their infection status [32]. This, to our knowledge, is one of very few studies to systematically evaluate population HBV and liver cancer-related awareness and knowledge in sub-Saharan Africa, and can contribute to locally-derived evidence to support HBV and liver cancer prevention efforts. Younger age in this study was associated with lower disease awareness and unrelated to comprehensive HBV knowledge, a finding that differs from a recent study in India [33], where younger people tended to have higher HBV knowledge. While this might be reflective of the younger generation access to information via technology in India, the observed lack of association in our population may be due to lower access to technology-transmitted knowledge than in India. Although about two thirds of the women knew about HBV prevention using a vaccine, a finding similar to that among Chinese pregnant women [34], a smaller proportion in this study was knowledgeable about mother to child HBV transmission and liver cancer as a possible consequence of chronic infection, compared to Chinese pregnant women. We also found a high proportion of women with inaccurate beliefs about HBV causation and transmission, compared to a Cameroonian study [28] in which prevalence of misconceptions was only 2.3%, but comparable to 66.5% of 504 pregnant women in a Ghanaian study [35]. Another important area where we found deficient knowledge was horizontal HBV transmission, where an infected adult may transmit to an infant through practices such as oral pre-warming or pre-chewing of infants’ food by adults, which may involve transmission through saliva or infected body fluids. Several studies [36–39] show evidence of horizontal HBV transmission through saliva, and this practice is common in developing country cultures [40–42], where it has been linked to HIV transmission, yet there is sparse data on knowledge of this route of HBV transmission among pregnant women. In cultures where these practices continue, specific attention will need to be given to effectively communicate horizontal route of transmission, as an effort to strengthen prevention. Both earlier [13] and more recent reviews [14, 15] of HBV transmission and prevention in different countries in SSA and East Africa region [16] did highlight the need to curb vertical and horizontal HBV transmission through provision of both routine HBV infant vaccination and neonatal HBV vaccination at birth, with detailed critique of existing challenges and opportunities therein. The scope of this discourse however, did not capture the wider community-level barriers, including HBV disease awareness or knowledge within the end-user communities, which this study has attempted to do.

Education was observed to be related to both HBV disease awareness and knowledge in this study. Most of our respondents had either a primary (31%) or a secondary (53%) education, with only 11% having a post-secondary education. The effect of education on HBV awareness was moderated by region of participants’ residence, with the result that for dwellers of the central region, having a secondary education conferred a 76% increase in prevalence ratios for HBV awareness, compared to only 25% increase, for northern region dwellers. This observation could be explained by the fact that in Uganda, HBV is more common in the north than the central region, and as such, residents of the north, regardless of their education level, are more likely to have heard about the disease through social networks of family and community than residents of the central region. Conversely, for residents of the central region, HBV disease awareness seems to be propagated more through years of formal literacy, than social networks, since the disease is less prevalent and as such, much less commonly reported in this region. The effect of years of formal education on comprehensive HBV knowledge, was not modified by region of residence. This finding is consistent with findings among pregnant populations within SSA [28, 43].

There were geographic differences in knowledge, with residents of northern region more likely to have heard of HBV, and to have adequate knowledge, and the association was consistent across the 3 models. HBV infection prevalence varies in Uganda, with the northern region having generally higher disease burden [6], which may explain higher odds of having heard of disease, despite significant deficiencies in disease-specific knowledge.

These findings have implications regarding the hepatitis control policy in Uganda and similar SSA settings; HBV prevention is done through HBV vaccination of infants as part of routine immunization. Interventions like antenatal HBV education, testing or vaccination for pregnant women, neonatal HBV vaccination at birth are lacking. This translates into gaps in HBV services for pregnant women and in locally-derived information such as HBV-status awareness among pregnant women. Comprehensive hepatitis education and accurate information will need to target the observed knowledge deficiencies and adequately attend to them.

This study had important strengths. Our method used multiple approaches to examine awareness and knowledge and we performed multiple sensitivity analyses to examine factors associated with disease awareness and knowledge, using an adequate sample size, to provide reliable estimates of awareness and knowledge, which can precisely inform HBV and liver cancer education interventions. Notable limitations of this work include the self-reported measures, as well as subjective measurement of disease awareness and knowledge which may carry inherent information bias. We attempted to minimize the effect of this subjectivity by constructing another variable that merged awareness of HBV disease and specific HBV knowledge, comparing those who scored “Low” on both with those who scored “high” on both, across a set of variables of interest.

## Conclusion

We have identified important knowledge deficiencies and misconceptions in relation to HBV among pregnant women. Even with geographical differences in disease awareness, both regions have critical information gaps that require adequate HBV prevention education policies, placing specific attention to women with fewer than 7 years of formal education, and adolescent pregnant women. Increasing HBV awareness and knowledge in obstetric populations is a critical component of HBV micro-elimination strategies that will lead to elimination of mother to child transmission of HBV by 2030. Accurately informed women may be more likely to seek and access testing and vaccination during pregnancy, as well as targeted birth dose vaccination for exposed newborns, if they have timely access to appropriate education messages. Therefore, designing education messages tailored to observed findings, in order to raise disease awareness and knowledge among pregnant women may be a key initial step towards micro-elimination in this territory.

## Abbreviations

HBV: Hepatitis B Virus
PR: Prevalence ratio.
SD: Standard deviation
SSA: Sub-Saharan Africa
WHO: World Health Organisation
